# PHOTO-STIMULATORY EFFECT OF LOW ENERGY HELIUM-NEON LASER IRRADIATION ON EXCISIONAL DIABETIC WOUND HEALING DYNAMICS IN WISTAR RATS

**DOI:** 10.4103/0019-5154.57606

**Published:** 2009

**Authors:** Arun G Maiya, Pramod Kumar, Shivanand Nayak

**Affiliations:** *From the Department of Physiotherapy, Manipal Medical College, Manipal, India.*

**Keywords:** *Clinical observation*, *hydroxyproline*, *laser photo stimulation*, *photo inhibition*, *wound healing*

## Abstract

**Background:**

Generally, the significances of laser photo stimulation are now accepted, but the laser light facilitates wound healing and tissue repair remains poorly understood.

**Aims:**

We have examined the hypothesis that the laser photo stimulation can enhance the collagen production in diabetic wounds using the excision wound model in the Wistar rat model.

**Methods:**

The circular wounds were created on the dorsum of the back of the animals. The animals were divided into two groups. The study group (N = 24) wound was treated with 632.8 nm He-Ne laser at a dose of 3-9 J/cm^2^ for 5 days a week until the wounds healed completely. The control group was sham irradiated.

**Result:**

A significant increase in the hydroxyproline content and reduction in the wound size were observed in the study group. The pro-healing actions seem to be due to increased collagen deposition as well as better alignment and maturation.

**Conclusion:**

The biochemical analysis and clinical observation suggested that 3-6 J/cm^2^ laser photo stimulation facilitates the tissue repair process by accelerating collagen production in diabetic wound healing.

## Introduction

Open wounds have lost the barrier that protects tissues from bacterial invasion and allow for the escape of vital fluids. Wound healing and tissue repair are complex processes that involve a dynamic series of events including clotting, inflammation, granulation tissue formation, epithelization, collagen synthesis, and tissue remodeling.[1]

The exact pathogenesis of the delayed wound healing is not clearly understood, but evidence from studies involving both human and animal models reveal several abnormalities in the various phases of the wound healing process. Impaired wound healing is an enigmatic and debilitating complication of diabetes and poses a serious challenge in clinical practice without expeditious healing, in which infections become more frequent. Most wound complications such as wound dehiscence or skin graft loss are associated with some form of host impairments such as infection, diabetes, or chemotherapy.[2,3]

In recent years, low-intensity laser therapy has gained considerable recognition and importance among treatment modalities for various medical problems including wound repair processes, musculo-skeletal complications, and pain control.[4,6] Clinical studies have shown low energy lasers to be effective as analgesics and to accelerate the healing of injured tissue.[7] Although the beneficial effects of laser photo stimulation are now generally accepted, the mechanisms by which laser light facilitates wound healing and tissue repair yet to be clearly understood.[8,9]

The Helium-Neon (He-Ne) laser has been used clinically on wounds to promote healing.[7] However, there are no enough data on the photo-stimulatory and/or photo-inhibitory effect of laser on the wound healing process. The efficacy of laser in wound healing remains un-established due to improper doses in the different phases of healing. The biochemical changes induced by low-level laser irradiation, and the mechanism by which laser facilitates healing of open skin wounds and resistant wound, like diabetic wound remains poorly understood. Therefore, the aims of the present study is to gain better insight into the photo-stimulatory and photo-inhibitory dose and effect of He-Ne laser light on wound healing, as this was not yet established with previous studies.

### Aim of the study

To study the photo-stimulatory effect of low energy He-Ne laser irradiation on excisional diabetic wound healing dynamics in Wistar rats.

## Materials and Methods

1. He-Ne laser with 632.8 nm wavelength (10 mW output, continuous mode, hand held probe with 1 cm^2^ spot area: EC 2000)
2. AutoCAD RL 14 Computer (AutoDesk Inc2000 USA)
3. Graph-paper
4. Drugs and surgical materials (alloxan, gloves, solvent ether, ketamine, syringes and saline obtained from Hospital Pharmacy)
5. Transparency sheets
6. The reagents for biochemical investigations (Lobachem chemicals, Germany)

### Procedure

The protocol of proposed work was submitted to the Institutional ethical committee and the clearance was obtained for the experiment on IAEC/KMC/July 2000.

### Animal selection and care

In-house bred albino Wistar strain male rats were used in the study. The range of weight of animals was between 170 and 250 g. All the animals were maintained for less than 12 hours day light environment. In each cage, only one animal was housed. For housing the animals, polypropylene cages (29 × 22 × 14 cm) were used with paddy husk bedding at 28° ± 1° and humidity of 55 ± 5%. The animals were kept in a hygienic environment and the bed was changed on alternate days. All the animals were provided with water and food *ad libitum*. The standard rat pellet food was supplied by Gold Mohur Lipton India Ltd. Breeding and maintenance of the animals were done as per the guidelines of Government of India for use of laboratory animals. (Government of India notifies the rules for breeding and conditioning animals experiments, proposed in the gazette of India, 1998; which was reproduced in *Indian Journal of Pharmacology*. 1999;31:92-5).

### Grouping

Seven groups depending on laser dose (3-9 J/cm^2^) were made, and 24 animals were present in each study and control groups [Table 1].

**Table 1:** Study and control groups were compared with different doses of laser exposure in diabetic wound healing

Group	Study group laser dose (J/cm^2^)	Control
I	3	No laser therapy
II	4	No laser therapy
III	5	No laser therapy
IV	6	No laser therapy
V	7	No laser therapy
VI	8	No laser therapy
VII	9	No laser therapy

### Induction of diabetes in Wistar rats by alloxan

The alloxan was injected intraperitonally to animals at 80 mg/kg body weight. While preparing alloxan, care was taken to maintain the alloxan solution at a very low temperature by placing ice cubes around the beaker, as alloxan is easily oxidized at room temperature. Alloxan was dissolved in 0.9% saline. Injection of alloxan was given to rats that had been deprived of food for about 24 hours. After about 30 minutes of injection, food was provided to animals *ad libitum*.

### Confirmation of diabetes and grouping

After a 7 days of stabilization period, blood samples were obtained from animals fasted overnight. Blood was drawn from intraorbital plexus by inserting a “mucocap” capillary. About 1-1.5 ml of blood was drawn into a test tube having sodium fluoride. The sample of blood was mixed with sodium fluoride and allowed to clot. After an hour, the sample of blood was centrifuged (R and C centrifuge) at 2000 rpm for about 15 minutes.

After centrifugation, the supernatant serum was collected and the glucose level was estimated. The alloxan-injected group with more than 200 mg% of blood glucose has been included in the study. The animals were assigned to the study and control groups, with —24 animals in each group with same weight, age, and blood glucose level. The day of confirmation of diabetes was taken as day 1 for further course of treatment as per the group. During the study period, the diabetic state of the animals was confirmed again by analyzing the blood glucose level periodically for both groups.

### Excisional wound procedures

#### Animals grouping

In diabetic wound healing study, the animals were assigned to the study and control groups with 24 animals in each group with comparable weight and age during different set of experiments.

#### Surgical procedure

The animals were anesthetized with intravenous ketamine of 2 mg/kg body weight. The dorsal furs of the animals were shaved with an electric clipper. The area of wound to be created was marked on the back of animals by methylene blue using circular stainless steel stencil. The full thickness of 4 cm^2^ excisional wound was created along markings using toothed forceps, a number 15 surgical blade and pointed scissors. Then, the area of the wound was recorded on a transparency paper. All the wounds were kept open. Each animal was kept in separate cage till the completion of the study, and the wounds of the study group (Laser) were healed due to secondary intention.

### Laser therapy protocol

The calibration and output of the equipment were checked before and during the experiment to maintain the accurate dosage with the help of a dosimeter. The method of irradiation was standardized before the experiment. The non-contact method (6-mm distance from the wound surface) was found to be accurate for irradiation in wound healing, and the technique was used during the study. In the study (laser) group for excisional wound a constant spot of 1 cm^2^ was irradiated for varying length of time to achieve the desired fluence or the dose. The treatment schedule of the study groups and control groups are shown in Table 1.

In each laser group the dosage was calculated using following formula:

D = p × t/A

D = Dose measured in J/cm^2^

p = Laser output in mW and it needs to be converted into Watts. In our equipment it has 10 mW output (divided by 1000 to convert to Watts) = 0.01 W

t = Treatment time in seconds

A = Area of the wound measured in cm^2^

During our present study, with different dose of laser and at different phases of healing, the time taken for irradiation of the wound was varied from 3 minutes to 27 minutes in the diabetic wound healing study.

### The experimental observation

The wounds were observed for the rate of contraction (healing), granulation tissue, and epithelization and mean wound healing time in both groups on different days.

The rate of contraction (healing) was calculated by following method:

Rate of contraction (R) = Initial (I) − Final area (F) × 100/ Number of days (N).

### Epithelization

To calculate the epithelization, the margin of the wound and the limit of epithelization were marked at the site of maximum and minimum epithelization, it was measured by vernier caliper, and the mean value of epithelialization was noted in square millimeter following complete healing in both groups.

### Granulation tissue

Granulation tissue was observed on seventh postoperative day in both groups. The granulation tissue graded subjectively depending upon the amount of granulation tissue present in both study and control groups was divided into four grades.

Grade I - no granulation tissue

Grade II - minimal granulation

Grade III - moderate granulation tissue

Grade IV - maximum granulation tissue

### Biochemical analysis: (Hydroxyproline level)

#### Sample collection and analysis

On fifth and final day, the granulation tissue of approximately 1 cm wide and 6 cm length was collected from each animal for analysis. The tissue was kept in an oven at 60°C and its dry weight was noted. Then 0.4 ml of deionized water, 1.0 ml of 2.5 N NaOH, 1.0 ml of 0.1 M CuSO_4_ and 1.0 ml of 6% hydrogen peroxide were added to 1 ml of hydrolysate. The tubes were covered and boiled for 15 minutes at 80°C, and absorbance was read at 540 nm using spectrophotometer to note the hydroxyproline level.

#### Method of statistical analysis

The experimental observation parameters (rate of contraction, epithilization, and mean wound healing time) between study and control groups were analyzed by independent ‘*t*’ test and expressed as mean ± SD.

The biochemical parameters (hydroxyproline) on day 5 and following complete healing were compared between study and control groups and analyzed by independent ‘*t*’ test and expressed as mean ± SD.

The significance level of the above results was predetermined at level *P* < 0.05. The SPSS-9 software package was used for statistical analysis.

## Results

The closure of skin defects was practically complete in all animals of the study group but varied in mean healing days in different dose of laser treatment. The wound healing process was significantly accelerated in groups exposed to He-Ne laser irradiation of 3-6 J/cm^2^ per day in the diabetic wound healing study. The significance was very much higher in the group irradiated with 4-5 J/cm^2^. At the doses between 7 and 9 J/cm^2^, slight deceleration was noticed in the healing process. In this study, the laser group's wound showed the formation of harder scabs at phase two of the healing process and in the final healing process. In all the different doses of experiment, the study and control groups were compared for the above mentioned parameters.

## Discussion

In an effort to provide a more clinically relevant wound-healing model, we investigated the effect of laser photostimulation on diabetic wound healing. The biochemical analysis and experimental observation results from this study clearly indicate that low energy photostimulation with He-Ne laser facilitate diabetic wound healing. In our study, we conducted a series of experiments on different dose of laser irradiation.

The photostimulatory effect was found in the dose between 3 and 6 J/cm^2^ in all the parameter like experimental observation and hydroxyproline level findings. The rate of contraction was significantly higher in the laser-treated group as compared to the control group with *P* < 0.0001 [Table 2a]. The epithelization was more in the study group as compared to the control group [Table 2b]. The mean wound healing time was significantly decreased in the study group of all the laser-treated group as compared to the control group with *P* < 0.0001 [Table 2c]. The subjective grading of granulation tissue on day 7 post-irradiation was significantly higher in the study group as compared to the control group [Table 2d]. This is supporting to the previous study,[10] however the study found that 4 J/cm^2^ was very effective in incisional wound healing, but the study was compared only for wound contraction rate and tensile strength. In our study, we have compared the parameters like experimental observation, area measurement, and hydroxyproline findings, and there was no single study reported, which conducted the experiment like our study. Our study showed the dose range between 3 and 6 J/ cm^2^ was effective, but the dose between 4 and 5 J/cm^2^ was found to be very effective in facilitating the wound healing process. Therefore, this dose can be considered as ‘therapeutic window’ in the wound healing process.

**Table 2:** Effect of 3-6 J/cm^2^ per day and 5 days a week dose of He-Ne laser radiation diabetic wound healing (a) Rate of contraction: Study versus control

Dose (J/cm^2^)	N	Study		Control		*t*	*P* value
					
		Mean	SD	Mean	SD		
3	24	3.36	0.79	1.98	0.45	5.21	0.0001
4	24	4.11	0.42	1.81	0.51	16.87	0.0001
5	24	4.16	0.52	1.58	0.47	17.86	0.0001
6	24	3.12	0.67	1.66	0.48	8.57	0.0001

**Table 2:** (b) Epithelization: Study versus control

Dose (J/cm^2^)	N	Study		Control		*t*	*P* value
					
		Mean	SD	Mean	SD		
3	24	2.29	1.04	1.45	0.65	3.31	0.002
4	24	4.62	0.87	1.87	0.74	11.74	0.0001
5	24	4.95	0.75	1.41	0.58	18.24	0.0001
6	24	3.70	0.69	1.70	0.46	11.77	0.0001

**Table 2:** (c) Mean wound healing time: Study versus control

Dose (J/cm^2^)	N	Study		Control		*t*	*P* value
					
		Mean	SD	Mean	SD		
3	24	31	1.91	53.41	2.70	−31.84	0.0001
4	24	25	1.62	54	3.50	−37.03	0.0001
5	24	23	1.30	55	1.41	−80.05	0.0001
6	24	30	1.35	55	1.75	−56.28	0.0001

**Table 2:** (d) Granulation tissue: On seventh post-operative day (grades): Study versus control group

Dose	N	Study				Control			
			
		I	II	III	IV	I	II	III	IV
3	12	02	04	04	02	06	06	-	-
4	12	-	04	04	04	05	07	-	-
5	12	-	05	05	02	06	05	01	-
6	12	-	04	02	06	05	07	-	-

Many studies[11–13] suggest that laser bio-stimulation occurs at fluences between 0.05 and 10 J/cm^2^, whereas fluences above 10 J/cm^2^ have inhibitory effects in the impaired wound healing process. Many of the chronic complication of diabetes involve defects in connective tissue such as poor wound healing.[13] The wound healing abnormalities of diabetes results from several causes.[14] When carbohydrates are unavailable to cells for normal aerobic metabolism, oxidation of amino acids for caloric needs results in amino acids and protein depletion. When glycogenolysis and gluconeogenesis fail to provide glucose to meet the energy requirements for fibroblasts and leucocytes, they become dysfunctional and impaired wound healing results. The poor wound healing of diabetic has been shown to be associated with a decreased amount of collagen fibrils and collagen production.[13] This study demonstrates that low energy laser enhances wound healing in diabetic rats as evidenced by experimental observation, area measurement, and biochemical and histopathological analyses of the study group and control group. In our study, we found that 3-7 J/ cm^2^ was found to be a stimulating dose, whereas increasing dose between 8 and 9 J/cm^2^ was a bio-inhibition dose in diabetic wounds.

In the present study, the influence of laser treatment on the healing process was most pronounced in biochemical findings. The results of the study show that the production of collagen in diabetic wounds can be modulated by laser treatment. The content of the total collagen was significantly increased in laser-treated wounds as compared to the control group. The total content of the collagen in the laser group was significantly more than that of the control group on day 5 and on healing in the group with 3-6 J/cm^2^ dose with *P* < 0.0001 [Table 3a and b]. In our study, we confirmed more production of collagen on the 5^th^ postoperative day in the study group as compared to the control group which indicates that collagen production can be further stimulated by laser irradiation. In diabetic wound healing, a higher dose like 7-9 J/cm^2^ showed deceleration in the wound healing process as evidenced by experimental observation such as the rate of contraction [Table 4a], epithelization [Table 4b], mean wound healing time [Table 4c], granulation tissue [Table 4d], the biochemical findings on day 5 and on complete healing [Table 5a and b].

**Table 3:** (a) Hydroxyproline (day 5): Study versus control

Dose (J/cm^2^)	N	Study		Control		*t*	*P* value
					
		Mean	SD	Mean	SD		
3	24	11.65	1.47	7.34	1.42	10.31	0.0001
4	24	11.86	2.04	6.45	1.21	11.13	0.0001
5	24	14.16	1.71	7.20	0.77	18.13	0.0001
6	24	13.08	1.28	6.75	0.98	19.15	0.0001

**Table 3:** (b) Hydroxyproline (on healing): Study versus control

Dose (J/cm^2^)	N	Study		Control		*t*	*P* value
					
		Mean	SD	Mean	SD		
3	24	28.59	4.33	12.09	1.99	16.95	0.0001
4	24	45.30	1.74	12.83	1.30	72.43	0.0001
5	24	47.75	2.80	13.41	1.47	53.15	0.0001
6	24	37.95	3.29	12.16	1.60	34.51	0.0001

**Table 4:** Effect of 7-9 J/cm^2^ per day and 5 days a week dose of He-Ne laser radiation diabetic wound healing (a) Rate of contraction: Study vs. Control

Dose (J/cm^2^)	N	Study		Control		*t*	*P* value
					
		Mean	SD	Mean	SD		
7	24	3.12	0.74	1.41	0.50	9.34	0.001
8	24	1.91	0.71	2.04	0.75	−0.59	0.558
9	24	1.20	0.41	1.20	0.41	0.000	1.00

**Table 4:** (b) Epithelization: Study versus control

Dose (J/cm^2^)	N	Study		Control		*t*	*P* value
					
		Mean	SD	Mean	SD		
7	24	3.29	0.75	1.66	0.48	8.92	0.001
8	24	1.58	0.50	1.54	0.50	0.285	0.777
9	24	1.08	0.28	1.12	0.33	−0.464	0.645

**Table 4:** (c) Mean wound healing time: Study versus control

Dose (J/cm^2^)	N	Study		Control		*t*	*P* value
					
		Mean	SD	Mean	SD		
7	24	38	3.99	55	2.85	−16.62	0.000
8	24	47	4.61	55	1.41	−7.91	0.000
9	24	49	0.65	55	0.85	−22.40	0.000

**Table 4:** (d) Granulation tissue: On seventh post-operative day (grades): Study versus control

Dose	N	Study				Control			
			
		I	II	III	IV	I	II	III	IV
7	24	13	8	03	-	15	06	03	-
8	24	14	10	-	-	16	08	-	-
9	24	12	10	02	-	13	10	01	-

**Table 5:** (a) Biochemical analysis: Hydroxyproline (day 5): Study versus control

Dose (J/cm^2^)	N	Study		Control		*t*	*P* value
					
		Mean	SD	Mean	SD		
7	24	6.66	0.56	6.41	0.65	1.41	0.163
8	24	5.20	0.58	6.25	0.53	−6.43	0.000
9	24	4.54	0.58	6.41	0.58	−11.08	0.000

**Table 5:** (b) Biochemical analysis: Hydroxyproline (on healing): Study versus control

Dose (J/cm^2^)	N	Study		Control		*t*	*P* value
					
		Mean	SD	Mean	SD		
7	24	11.54	1.69	11.62	1.09	−0.202	0.841
8	24	9.66	0.63	9.37	0.71	1.49	0.141
9	24	9.41	0.82	9.08	0.82	1.39	0.171

The mechanism by which laser photostimulation facilitates collagen production in diabetic wound healing was not clear with the previous study. This effect may involve a variety of photo-stimulating mechanisms. It is mainly because the laser energy at certain frequencies can modulate cell proliferation and release the growth factors from fibroblasts. The other mechanism of photostimulation was that the mitochondria are the photoacceptors for light energy. The absorption of energy by the respiratory chain may cause oxidation of NADH, producing changes in the redox status in mitochondria and cytoplasm. The activation of electron transport chain results in an increase in the electrical potential across the mitochondria membrane, an increase in the ATP pool, and finally the activation of nucleic acid synthesis. It also enhances the pro-collagen production, increased cross-linking of existing collagen molecules, acceleration of epithelial repair, and early growth of granulation tissue.[21] In our study, both biochemical findings of 3-7 J/cm^2^ showed significant difference in the study group as compared to the control group with *P* < 0.0001.

The magnitude and the specificity of changes in collagen production in diabetic wounds demonstrate that collagen is more responsive to the altered conditions of diabetes than was previously suspected. Within 2 weeks of the onset of diabetes, collagen production was decreased to 50% compared to that of the non-diabetic group. With higher plasma glucose levels, there was a lower relative rate of collagen production in tissue and at every level of hyperglycemia; collagen was decreased to a greater degree than non-diabetic tissue. Previous studies confirm our observation of decreased collagen production in diabetes. Diabetic animals have been shown to have poor wound healing and this has been associated with a decreased amount of collagen.[13] In addition to defects in collagen production, posttranslational modifications of the collagen peptide have been also been reported to occur in diabetes. Therefore, posttranslational modifications of collagen in diabetes may lead to excess collagen accumulation under conditions where there is decreased synthesis of collagen. Thus, our studies in combination with others suggest that there is defect in collagen metabolism, decrease in collagen production and altered posttranslational modifications of collagen that affect the turnover of the collagen in diabetes.

In our study, we found that the low level He-Ne laser is found to be effective in diabetic wounds. The results of the experimental observation, biochemical findings of 3-7 J/ cm^2^ dose (photo-stimulation dose) of the present study showed that the laser-treated group was healed better and faster with *P* < 0.0001 as compared to the control group, and an increasing dose between 8 and 9 J/cm^2^ was found to decelerating the reparative process and hence called a bio-inhibiting dose in diabetic wound healing.

The review of the literature provides the considerable contradicting data on low energy laser bio-stimulation of wound healing.[15] *In vitro* experiments support the hypothesis that low energy laser irradiation may accelerates wound healing.[16] Demonstration of increased collagen synthesis *in vitro* suggests that tensile strength of incisional wounds might be increased in treated wounds. Studies that show that He-Ne irradiation produce a massive transformation of fibroblasts into myofibroblasts[17] suggest the possibility of an increased rate of wound contraction in response to laser treatment. Although these cellular and biochemical events are well documented, the application of this knowledge to wound healing acceleration has been frustrated by equivocal reports.[9] Kana *et al*.[10] succeeded in demonstrating accelerated healing in an incisional model, and found significant differences in an excisional model. Mester *et al*.[5] has produced positive results in both models, whereas several authors have reported only negative results. Strikingly, both Braverman *et al*.[18] and Surinchak *et al*.[19] independently reported increased tensile strength in an incisional model, but failed to produce increased contraction rates in an excisional model.

However, there is always the question of incorrect or inadequate dosage, and unsuitable indications for low level laser therapy (LLLT). By far the majority of reports appearing in the literature describe at least positive effects of LLLT, in clinical and experimental studies in animals.[20] Keeping all the laser characteristics, wave length, coherence, linear polarization, and pulse frequency constant, we used various dose schedule of the He-Ne laser and achieved a bio-simulative effect at (3-6 J/cm^2^) doses or gradually leads to bio-inhibitory effect with increasing doses (7-9 J/cm^2^). In the present study, we found that the laser energy dose between 3 and 6 J/cm^2^ was found to be bio-simulative dose, whereas the laser dose above 6 J/cm^2^ is found to have bio-suppressive or inhibition effect with increasing doses (7-9 J/cm^2^).

## Mechanism of biological effect of low energy laser therapy

The low energy at wavelength of 632.8 nm can modulate the cell proliferation and the release of growth factors from fibroblasts.[21] Therefore, the positive effect of laser photostimulation on wound healing may involve the enhancement of growth factor release, which in turn promotes extracellular matrix production and degradation. In our study, we found that the main biological effect of laser is due to the major absorbing structures. The major absorbing structures for the red visible laser wavelengths are the proteins; however, the identity of the photoreceptors responsible for the biological effects of low energy laser therapy (LELT) has not been resolved. Several studies have suggested that either elements in the mitochodrial cytochrome system or endogenous porphyrins in the cells are the energy-absorbing chromophores in LELT.[22]

The other mechanism could be due to the reason that, laser light affects the mitochondrial respiratory chain by changing the electric potential of cell membranes and consequently their selective permeability for sodium, potassium, and calcium ions or by the increasing the activity of certain enzymes such as cytochrome oxidize and adenosine triphosphatase. It also increases DNA synthesis, collagen, and procollagen production and may increase cell proliferation or alter locomotors characteristic of the cells. This findings in our study was also supported by a similar finding of the previous report.[23,17] The result of this study and the possible biomechanics involved are discussed in the context of other experimental findings of increased cell counts following —He-Ne radiation.[26] The present study suggests that photoenergy of 632.8 nm wavelength at the given parameters possibly induced the fibroblasts to secrete the growth factors that probably acted in an autocrine manner to increase their rate of mitosis and or reduce cell death.

The response of low energy laser on cells may be dose dependent[16] as well as wavelength dependent.[24] Therefore, we strongly suggest that correct energy density with an appropriate wavelength can be absorbed by the targeted tissues. Therefore, employing the correct power density, exposure time, and energy density are important parameters to achieve the bio-stimulation effect in wound healing.

In our study, we found that the reason for the bio-inhibition mechanism with increasing doses was due to inadequate incident photoenergy or had exceeded the stimulatory range for inducting stronger biological activities in the cells. The inhibition with higher dose was probably caused by formation of dry harder scar. For this effect, absorption of light energy in the tissue in the form of heat, i.e. without wavelength specificity, seems to be of importance. However, the higher dose leading to direct thermal destruction of tissues was described earlier after long-term irradiation.[25] Thus, the excessive cumulative effects of daily exposure to low energy laser for several days may have reversed any initial beneficial effects of red light irradiation that also occurred in few previous studies.[23]

In our study, we found that the low level He-Ne laser is found to be effective in open skin excisional wounds. The results of the experimental observation and biochemical findings of 3-6 J/cm^2^ dose (photo-stimulation dose) of the present study showed that the laser-treated group were healed better and faster with *P* < 0.0001 as compared to the control group, and an increasing dose between 7 and 9 J/ cm^2^ was found to decelerating the reparative process and hence called a bio-inhibiting dose in diabetic wound healing.

## Conclusion

We attempted to establish an excisional model of low energy bio-stimulation and bio-inhibition dose of laser on wound healing.

- In diabetic wound healing, the experimental observation (rate of contraction, epithelization, mean wound healing time) of the study group exposed to 3-7 J/cm^2^ dose were highly significant as compared to the control group (*P* < 0.0001).
- The granulation tissue was significantly increased in the study group of 3-7 J/cm^2^ as compared to the control group.
- The hydroxyproline level of the study group exposed to 3-7 J/cm^2^ doses was significantly higher as compared to the control group in the diabetic wound healing process (*P* < 0.0001).
- An increasing dose between 7 and 9 J/cm^2^ was found to decelerating the reparative process and hence called bio-inhibiting dose in diabetic wound healing.
- Therefore, we concluded that low energy —He-Ne laser irradiation at certain dose has a significant beneficial effect on wound healing. The present study highlights the possible utility of He-Ne laser with appropriate energy density as an adjunctive modality for diabetic wound healing in clinical practice.
